# Novel mutation sites in the *Bubaline MC1R* gene

**DOI:** 10.5455/javar.2025.l994

**Published:** 2025-12-25

**Authors:** Widya Pintaka Bayu Putra, Hikmayani Iskandar, Tulus Maulana, Ekayanti Mulyawati Kaiin, Syahputra Wibowo, Erni Damayanti, Syahruddin Said

**Affiliations:** 1 Research Center for Applied Zoology, National Research and Innovation Agency (BRIN), Bogor, Indonesia; 2 Eijkman Research Center for Molecular Biology, National Research and Innovation Agency (BRIN), Bogor, Indonesia; 3 Department of Animal Production, Faculty of Animal Science, Hasanuddin University, Makassar, Indonesia

**Keywords:** *Bubalus bubalis*, *MC1R* gene, coat color, nucleotide variation, protein structure, sequencing

## Abstract

**Objective:**

Buffalo (*Bubalus bubalis*) is an important livestock species raised for meat, milk, and draught purposes. In Indonesia, buffaloes with rare coat colors (e.g., white, striped) hold cultural significance, especially in Toraja funeral traditions. This study aimed to identify mutation sites in the exon 1 region (822 bp) of the *Melanocortin 1 Receptor* (*MC1R*) gene in buffaloes using forward sequencing.

**Materials and Methods:**

Four Toraya buffaloes (1 white, 2 striped, and 1 black) and two black Murrah buffaloes were used as experimental animals. In addition, seven *MC1R* gene sequences from different buffalo breeds (Murrah × Dehong (light grey), Dehong (white and dark grey), Murrah (black), Jafarabadi (black), and Surti (brown)) were obtained from the NCBI database for comparative analysis.

**Results:**

A total of five nucleotide variation sites were identified in the experimental animals, including three novel mutations (c.26M, c.49Y, and c.50R) and two previously reported mutations (c.170R and c.244K). Among these, c.50R was identified as a synonymous mutation, while the remaining mutations were non-synonymous and predicted to affect the amino acid sequence of the MC1Rprotein. Notably, all three novel mutation sites were consistently present in all studied Toraya and Murrah buffaloes, suggesting shared genetic variants across phenotypically distinct populations. Structural prediction analysis indicated that these mutations could potentially alter the conformation and function of the MC1R protein.

**Conclusion:**

The identification of three novel mutations in the *MC1R* gene enhances our understanding of coat color variation and genetic diversity in Indonesian buffalo populations, particularly those of cultural and economic significance.

## Introduction

Buffalo (*Bubalus bubalis*) is an important livestock species for various purposes, including meat and milk production, as well as draught resources. Despite this, the buffalo with a rare coat color was used in the funeral tradition by the Toraja ethnic group in Indonesia [1]. In this tradition, the Toraja buffaloes must be sacrificed to accompany the funeral procession. Toraya buffaloes exhibit a variety of coat color types, includingwhite, black, and striped [2]. Currently, the Toraya buffalo has been designated as one of the Indonesian native buffalo breeds, as per the decision of the Indonesian Ministry of Agriculture No. 2845/Kpts/LB430/8/2012 [2]. According to the mitochondrial D-loop gene, the Toraya striped buffalo is classified in a different cluster and separated from buffalo populations from Sumatra, Java, and West Nusa Tenggara [3].

Previously, Yusnizar et al. [4] reported a novel mutation site of c.328Y in the exon 3 region of the *Microphthalmia-associated transcription factor* (*MITF*) gene that is associated with white-spotted coat color in Toraya buffalo. Nonetheless, many studies reported that *Melanocortin 1 receptor* (*MC1R*) gene also influences the coat color patterns in buffalo [5,6]. In contrast, Chen et al. [7] and Jakaria et al. [8] reported no association between the *MC1R* gene polymorphism and coat color trait in yak (*Bos grunniens*) and Bali cattle (*Bos javanicus*), respectively. In cattle (*Bos taurus*), the *MC1R* gene is located on chromosome 18 along 1751 bp (GenBank: NC_037345.1) with an exon along 954 bp [9]. The *MC1R* gene plays a vital role in melanogenesis and determines the coat color of mammals [10]. The *MC1R* gene encodes a melanocytic Gs protein-coupled receptor that is integral to the regulation of skin pigmentation, UV radiation response, and melanoma susceptibility. This gene exhibits high polymorphism, and loss-of-function variants are correlated with phenotypes characterized by fair skin, heightened UV sensitivity, and an elevated risk of melanoma. The underlying mechanisms for these associations involve defective epidermal melanization and compromised DNA repair processes [11]. Proteins are intricate macromolecules that play essential roles in nearly all critical biological processes within an organism, such as metabolism, molecular transport, signal transduction, and various other functions [12].

Unfortunately, the detection of the *MC1R* gene polymorphism among Indonesian buffaloes with different coat colors has not been reported. The current study aimed to detect mutation sites in the *bubaline MC1R* gene and their effect on the protein structure of *MC1R*.

## Materials and Methods

### Ethical approval

The Animal Ethics Committee of the National Research and Innovation Agency (BRIN) has approved this study with certificate number 093/KE.02/SK/05/2023, regarding the use of animal models and experimental design.

### Animals and DNA extraction

**Figure 1.**
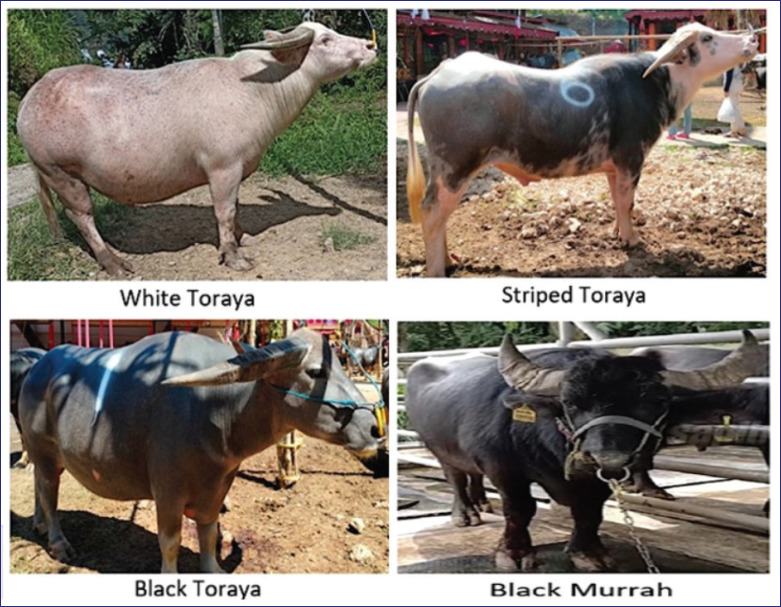
The patterns of coat color in Toraya and Murrah buffaloes.

Four Toraya buffaloes (1 white, 2 striped, and 1 black) and two black Murrah buffaloes were used for the experimental animals in the present study (Fig. 1). The Toraya buffaloes (swamp type) in this study originated from Toraja Regency. While the black Murrah buffaloes (river type) were used as control animals in the present study and originated from the National Artificial Insemination Center (NAIC) in Lembang, West Java. Therefore, 5 ml of blood samples were collected from the* jugular vein* of each animal. The DNA extraction was performed using a genomic DNA extraction kit (Geneaid, Taiwan) and stored at −20°C for further analysis.

### PCR and sequencing

The amplification of the *bubaline* *MC1R* gene (exon 1) was performed in a total volume of 30 µl consisting of 9 µl of DNA template, 0.6 µl of each primer, 15 µl of GoTaq Green PCR Master Mix (Thermo Scientific, USA), and 4.8 µl of nuclease-free water. An 822 bp of *MC1R* gene sequence (GenBank: AF445641) was amplified using the primer pairs of Forward: 5’- CAT CCC TGA CGG GCT CTT TCT C -3’ and Reverse: 5’- AGC ACC TCT TGG AGC GTC TTC C -3’ [5]. The *bubaline MC1R* gene amplification was performed in a thermocycler machine (Applied Biosystem, USA) with the PCR program consisting of 1 cycle of pre-denaturation at 95°C for 2 min, followed by 35 cycles of denaturation at 95°C for 1 min and 30 sec, annealing at 60°C for 1 min, initial extension at 72°C for 1 min, and final extension at 72°C for 5 min. Electrophoresis was performed using a 1% agarose gel stained with 2 μl of SYBR Safe DNA Gel Stain (Invitrogen, USA) for 30 min at 110 volts. The DNA visualization was captured using the G-box Documentation System (Syngene, UK). The sequencing analysis was performed by 1st BASE Laboratory Service (Malaysia). Therefore, the alignment analysis was performed using the BioEdit package to detect mutation sites in the target sequence [13].

### Protein sequence alignment

Sequence alignment and visualization of conserved amino acids were performed using the COBALT constraint-based multiple protein alignment tool (https://www.ncbi.nlm.nih.gov/tools/cobalt/re_cobalt.cgi) [14]. The universal protein resource (Uniprot) (http://www.uniprot.org/align/) [15], utilizing the default parameters for both tools [16,17].

### MC1R protein structures

The three-dimensional structures of both the wild-type and mutant *MC1R* proteins were generated using AlphaFold3 (https://deepmind.google/technologies/alphafold/). These modelled structures were then utilized for comprehensive structural analysis, and their structural quality was analyzed by using PDBsum (https://www.ebi.ac.uk/thornton-srv/databases/pdbsum/). Then, it was analyzed for its secondary structure details [18].

## Results and Discussion

**Figure 2.**
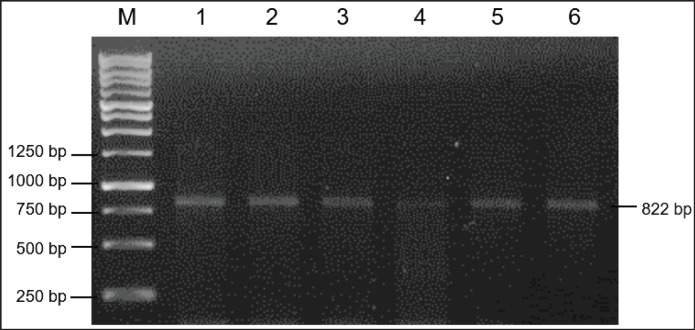
Amplification of the* bubaline MC1R* gene. Line 1-6: DNA sample; M: DNA ladder 250 bp.

**Figure 3.**
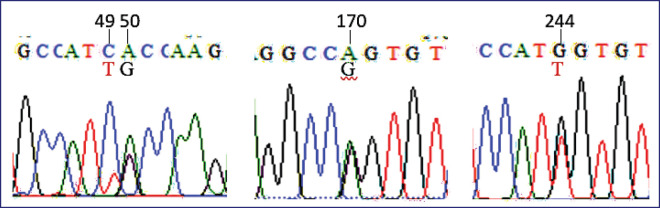
Detection of mutation sites in the *MC1R* gene.

Along 822 bp of the amplicons, primer pairs were used according to the* B. taurus MC1R* gene (GenBank: AF445641), as shown in Figure 2. Therefore, five mutation sites were detected in the target sequence of the *bubaline MC1R* gene, i.e.,c.26M, c.49Y, c.50R, c.170R, and c.244K (Fig. 3). Interestingly, three mutation sites of c.26M, c.49Y, and c.50R have not been reported in many previous studies. While two mutation sites of c.170R and c.244K are two common mutation sites in the *bubaline MC1R* that also occurred in Murrah cross buffalo (GenBank: GQ359897), as shown in Table 1. In addition, the mutation site of c.26M was identified as the genetic marker to discriminate between Toraya/Australian Murrah and buffaloes from China and India. Regarding the common mutation sites (c.170R/c.244K), buffalo coat colors can be associated with specific genotypes: EBS/EBS or GG/GG (grey color), EBR/EBR or AA/TT (black, white, and brown colors), and EBRS/EBRS or AA/GG (grey color) genotypes [5,6]. In Toraya and Murrah buffaloes, both loci were monomorphic, exhibiting heterozygous alleles. However, the novel mutation of c.49Y was found to be polymorphic in Toraya buffaloes. Notably, a genetic marker associated with coat colors in Toraya buffaloes was not detected in the* MC1R* gene. Although synonymous mutations do not alter the amino acid sequence, they may affect gene expression or mRNA stability. In this study, synonymous mutations such as c.26M may serve as molecular markers rather than causal mutations for coat color. Further investigation with larger populations is necessary to confirm the consistency and reliability of these phenotypic markers.

**Table 1.** Detection of mutation sites in the *MC1R* gene of Toraya striped buffaloes.

Buffalo	GenBank	Coat color	Origin	Position* (exon 1)					Reference
				26	49	50	170	244	
Murrah × Dehong	GQ359897	Light grey	China	A	C	G	R	K	[5]
Dehong	GQ359954	Dark grey	China	A	C	G	G	G	[5]
Dehong	GQ359870	White	China	A	C	G	G	G	[5]
Murrah	MN687828	Black	India	A	C	G	A	T	[19]
Jafarabadi	MF421425	Black	India	A	C	G	A	T	[9]
Nili Ravi	MF421522	Black	India	A	C	G	A	T	[9]
Surti	MF421443	Brown	India	A	C	G	A	T	[9]
Toraya 1	LC844121	White	Indonesia	C	Y	R	R	K	Present study
Toraya 2	LC844122	Striped	Indonesia	C	C	R	R	K	Present study
Toraya 3	LC844123	Black	Indonesia	C	Y	R	R	K	Present study
Toraya 4	LC844124	Striped	Indonesia	C	Y	R	R	K	Present study
Murrah 1	LC844125	Black	Australia	C	Y	R	R	K	Present study
Murrah 2	LC844126	Black	Australia	C	Y	R	R	K	Present study

*Nucleotide position at GenBank: GQ359897; Y = pyrimidines (C or T); R = purines (A or G); K = keto bases (G or T)

**Figure 4.**
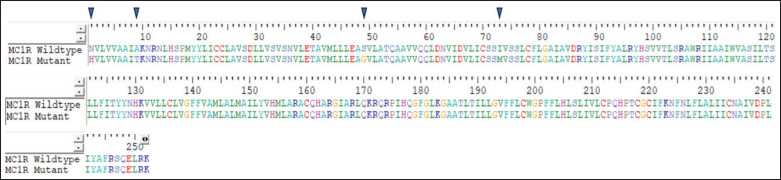
Four amino acid changes (arrow) in the two variants of the* bubaline MC1R* gene.

The five identified mutation sites in the *bubaline MC1R* gene are predicted to result in alterations to four amino acids, i.e.,p.N1H (c.26M), p.A9T (c.50R), p.S49G (c.170R), and p.I73M (c.244K), as shown in (Fig. 4). The sequence alignment of the *bubaline MC1R* gene identifies four distinct amino acid substitutions between the wild-type and mutant variants, marked by arrows at positions 10, 20, 40, and 70. These point mutations involve the replacement of native amino acids with different residues, potentially inducing significant alterations in the three-dimensional conformation and functional dynamics of the *MC1R* protein. In cattle, coat colors are determined by the presence of different pigments: eumelanin results in black coloration, while pheomelanin leads to red coloration, which are essential in the melanogenesis pathway and ultimately result in varying coat colors [9]. Variations in the *MC1R* are known to affect coat color across buffaloes [5]. Such modifications are likely to affect the receptor’s ligand-binding affinity and the efficacy of downstream signal transduction pathways. Consequently, these structural changes may contribute to variations in pigmentation phenotypes and other associated traits in the *bubaline* species. This detailed molecular analysis enhances our understanding of the genetic basis underlying phenotypic diversity.

**Figure 5.**
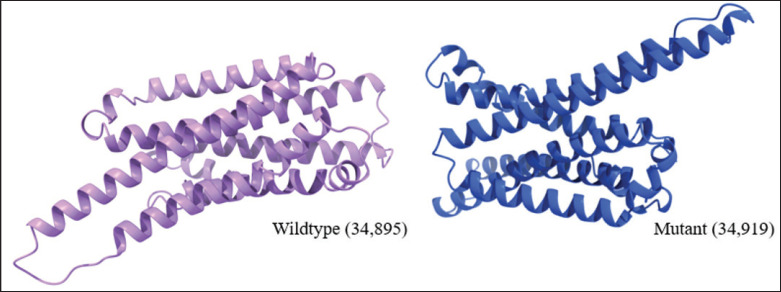
Two different protein structures of *bubaline MC1R*.

AlphaFold 3 utilized deep learning algorithms to predict the protein structure of the MC1R, based on the amino acid sequences of its subunits. Four amino acid changes were able to create the different MC1R protein structure form (Fig. 5), showing the 3D structural models of the bubaline MC1R protein, comparing the wild-type (34,895 kDa) and mutant (34,919 kDa) variants. Both structures were generated using homology modeling. Our *in silico* dimerization of both wild-type and mutant forms indicates that the presence of ARG 174 in the disallowed region suggests that this residue may inherently face steric constraints due to its local environment. However, the addition of GLN 157 in the disallowed region in the mutant form indicates a mutation-induced structural strain, consistent with the finding that mutations can affect protein stability and function [19].

The *MC1R* gene has demonstrated that mutations can disrupt protein stability and function through unfavorable interactions, a phenomenon commonly observed in mutation studies [20]. The overall lengths of the helices exhibit slight variations between the wild-type and mutant proteins. For instance, in Helix 3 (Pro17-Glu47), the length measures 46.99 Å in the wild type but decreases slightly to 46.92 Å in the mutant. Similarly, the unit rise, which represents the vertical distance between residues along the helical axis, also shows subtle differences. In Helix 4 (Ala55-Phe92), the unit rise increases from 1.49 Å in the wild type to 1.50 Å in the mutant, reflecting a minor adjustment in the helix structure. One of the most significant differences lies in the deviation from ideal geometry. For Helix 3, this deviation increases from 11.1 in the wild type to 13.5 in the mutant, indicating a potential distortion in the helix. Helix 4 also experiences a slight change, with deviations shifting from 19.4 in the wild type to 19.1 in the mutant.

The distances between interacting helices show slight shifts in several cases. For example, the distance between helices A1 and A3 in Interaction 1 decreases from 8.9 Å in the wild type to 8.5 Å in the mutant, indicating closer packing. Similarly, in Interaction 3 (A3 and A4), the distance changes minimally, from 8.2 Å in the wild type to 8.3 Å in the mutant. Alongside these changes in distance, the angles of interaction also exhibit subtle variations. In Interaction 1 (A1 and A3), the angle shifts from −146.3° in the wild type to −143.5° in the mutant, while in Interaction 10 (A4 and A9), it moves slightly from −153.1° to −153.5°. The number of residues involved in these interactions also varies slightly. For instance, in Interaction 3 (A3 and A4), the mutant involves 30 residues (15 from each helix), compared to 31 residues (16 from A3 and 15 from A4) in the wild type. This reflects minor differences in residue participation. In Interaction 16 (A9 and A10), the total number of interacting residues remains the same (6), though their distribution between the helices differs slightly. These nuanced changes collectively highlight subtle structural adjustments in the mutant protein.

The beta turns in the mutant protein differ noticeably from those in the wild type. One key difference is the reduction in the number of beta turns, dropping from nine in the wild type to just five in the mutant. This loss suggests that certain beta-turn regions are missing, which could influence how the protein folds and its overall compactness. Some beta turns are still present in the mutant, but they show small differences. For example, Turn 1 (Ser49-Ala52) in the mutant matches Gly49-Ala52 in the wild type, except for the first residue, which changes from glycine to serine. Meanwhile, Turn 5 (Ala244-Ser247) is retained in both versions, but there are slight shifts in its conformational angles. Interestingly, the types of these shared beta turns remain consistent. Turn 1, for instance, is still classified as type VIII, while the others are classified as type I, indicating that their structural roles have not changed drastically. Subtle adjustments in the angles of certain residues also highlight structural changes. In Turn 2 (Tyr127-His130), the Phi angle for residue i+1 shifts slightly from −58.1° in the wild type to −53.2° in the mutant, while the Psi angle for residue i+2 moves from −68.4° to −66.1°. Turn 4 (Tyr243-Arg246) sees a more notable change, with the Phi angle for residue i+1 moving from −85.8° to −78.6°.

Then the compactness of the beta turns, measured by the distance between residues i and i+3, stays mostly unchanged. Turn 1, for example, retains a consistent distance of 6.0 Å in both forms, suggesting that its packing remains similar. However, Turn 4 becomes slightly less compact, with the distance increasing from 5.8 Å in the wild type to 5.9 Å in the mutant. In addition, hydrogen bonding changes are evident, with Turn 4 regaining a stabilizing bond in the mutant that was absent in the wild type. These differences, although subtle, suggest that the mutant protein has undergone changes in its beta turns that could impact its overall stability and function. While some regions remain structurally similar, the loss and adjustment of specific beta turns reflect the impact of the mutation.

The activation level of the* MC1R* receptor is primarily influenced by three key factors: the concentration of extracellular agonists, such as α-MSH (alpha-melanocyte stimulating hormone), which stimulate receptor activity; the concentration of extracellular antagonists or inverse agonists, such as Agouti signaling protein (ASIP), which inhibit receptor activity; and the receptor’s intrinsic basal activity, which reflects its inherent level of activation in the absence of external stimuli [21]. The *MC1R* gene encodes a G-protein-coupled receptor primarily involved in regulating skin pigmentation by controlling melanin production in melanocytes [11]. Structural alterations, such as mutations leading to amino acid substitutions, can significantly impact the receptor’s function. For instance, changes in stability or folding could impact its ability to interact with other molecules or perform its biological role effectively [22]. Further studies are essential to elucidate the precise functional consequences of these structural changes and their broader biological implications.

**Figure 6.**
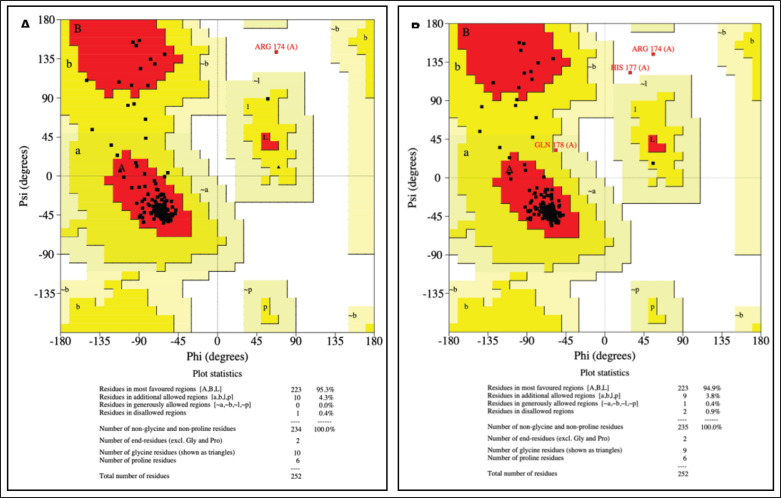
Ramachandran plot of *MC1R* gene.A. Wildtype. B. Mutant.

The Ramachandran plots of the *MC1R* gene for the wild-type and mutant forms (Fig. 6) were analyzed to assess the impact of the mutation on the protein’s structural stability. In the wild-type structure, 223 residues (95.3%) were located in the most favored regions, while in the mutant structure, this number slightly decreased to 222 residues (94.9%). This reduction indicates a minor shift in structural stability introduced by the mutation. Additionally, the number of residues in the additional allowed regions remained constant at 10 residues (4.3%) for both forms, suggesting that the overall structural flexibility was not significantly altered. While the Ramachandran plot provides a static overview of structural quality, understanding the dynamic effects of mutations in the *MC1R* gene, such as changes in stability or signaling efficiency, requires molecular dynamics simulations [23]. Future research could incorporate molecular dynamics simulations to validate these findings and assess the effects of the mutation under biologically relevant conditions. Additionally, experimental techniques such as X-ray crystallography or NMR spectroscopy are essential for validating computational findings and elucidating mutation-induced structural differences [24,25].

**Figure 7.**
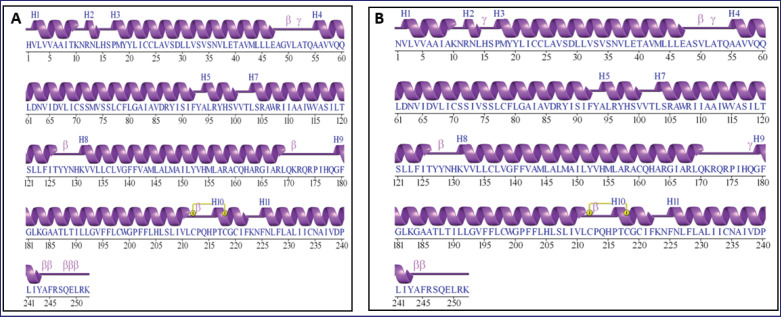
Secondary structure of *MC1R*.A. Wildtype. B. Mutant.

The presence of residues in disallowed regions increased in the mutant structure. In the wild type, only one residue (0.4%) was found in disallowed regions, specifically Arg 174. However, in the mutant form, two residues (0.8%) occupied disallowed regions, namely ARG 174 and GLN 157. The addition of GLN 157 in the disallowed region highlights the potential destabilizing effect of the mutation on the protein structure. These differences suggest that the mutation may introduce localized strain or steric clashes. The secondary structure of *MC1R* in wild-type and mutant forms: it was observed that the structures generated by both computational methods are not significantly different from the native structure (Fig. 7).

The novel mutation sites in the *bubaline MC1R* gene will help us to understand coat color variation in buffalo. By elucidating the molecular mechanisms governing pigment production, these findings provide critical insights into genetic diversity. Understanding these genetic variations not only enhances our knowledge of coat color inheritance but also sheds light on the broader implications for the adaptive strategies and domestication processes of this economically and culturally significant species. Furthermore, this research highlights the importance of comprehensive genetic studies that utilize advanced sequencing techniques and diverse sample sources to unravel the complexities of genetic diversity in livestock and related species. While this study provides insights into the genetic variation of *MC1R* and its potential association with coat color in Bali cattle, the comparative context with other species remains limited. Previous research has shown that *MC1R* mutations are strongly associated with coat color phenotypes in species such as buffalo and pigs. However, similar variants in yak or Bali cattle do not always correlate with observable changes in pigmentation. This discrepancy may be attributed to species-specific differences in melanogenesis pathways, gene–gene interactions (e.g., with *ASIP,* *MITF*, or *TYRP1*), or regulatory mechanisms that compensate for *the disruption of* *MC1R*. A more comprehensive cross-species comparative analysis, including gene expression profiling and epistatic interaction studies, is needed to elucidate these divergent phenotypic outcomes. Another limitation lies in the exclusive reliance on *in silico* predictions to assess the potential impact of nonsynonymous SNPs on *MC1R* protein function. Although computational tools such as PolyPhen-2 and PROVEAN provide preliminary indications of deleterious effects, these predictions do not substitute for experimental confirmation.

## Conclusion

The identification of five mutation sites in the *bubaline MC1R* gene has significantly enhanced our understanding of its structural and functional dynamics. Among these, three mutations are novel, with their predicted amino acid substitutions indicating potential effects on protein stability and function. Moreover, the findings demonstrate that these mutations alter the *MC1R* protein structure, particularly affecting alpha helices and beta turns, which in turn influence phenotypic traits.

## List of abbreviations

α-MSH, alpha-melanocyte stimulating hormone; μl, microliter; ASIP, Agouti signaling protein; MC1R, *Melanocortin 1 Receptor*; MITF, *Microphthalmia-associated transcription factor*; ml, milliliter; min, minutes; sec, seconds.
